# Case Report: Pulmonary tuberculosis with pneumocystis jirovecii colonization in a non-HIV patient: a cautionary tale on interpreting mNGS results

**DOI:** 10.3389/fmed.2026.1782843

**Published:** 2026-04-09

**Authors:** Qikai Chen, Zheng Yang, Danhong Ren, Chunhua Bao, Yi Zhao, Zhanli Shi

**Affiliations:** 1 The Second School of Clinical Medicine, Zhejiang Chinese Medical University, Hangzhou, Zhejiang, China; 2 Department of Intensive Care Unit, Hangzhou Red Cross Hospital, Hangzhou, Zhejiang, China; 3 Department of Radiology, Hangzhou Red Cross Hospital, Hangzhou, Zhejiang, China; 4 Department of Pathology, Hangzhou Red Cross Hospital, Hangzhou, Zhejiang, China

**Keywords:** case report, diagnosis, metagenomics next-generation sequencing (mNGS), *Mycobacterium tuberculosis*, pathogens, pneumocystis jiroveci pneumonia

## Abstract

The diagnosis and treatment process of this case highlights that mNGS, as a powerful pathogen detection tool, provides a rapid method for the early detection of Pneumocystis jirovecii. However, mNGS testing of lavage specimens alone cannot distinguish between colonization and infection by the pathogen, particularly when a high number of sequences are present. Clinicians should therefore interpret laboratory results with caution to avoid unnecessary treatment that may cause adverse effects to the patient. CT scans offer strong evidence for differentiating between Pneumocystis jirovecii infection and/or *Mycobacterium tuberculosis* infection. Performing a biopsy at the site of infection, collecting pathological samples, and submitting them for mNGS testing can further assist clinicians in making a definitive diagnosis.

## Background

Pneumocystis jirovecii and *Mycobacterium tuberculosis* are common opportunistic pulmonary pathogens in patients infected with human immunodeficiency virus (HIV). However, co-infection with these two pathogens is exceedingly rare among HIV-seronegative patients. Using the Foreign Medical Literature Retrieval System (FMRS) provided by Metech Innovation Co., Ltd. in Shenzhen, China, we conducted a search with “Pneumocystis jirovecii” and “tuberculosis” as titles or subject terms, yielding only 5 reported cases.

With the widespread application of metagenomic next-generation sequencing (mNGS) technology, the clinical detection rates of uncommon pathogens such as Pneumocystis jirovecii have significantly increased. This advancement holds crucial value for early diagnosis of both pneumocystosis and *Mycobacterium tuberculosis* infections in HIV-seronegative patients. Nevertheless, the exceptional sensitivity of mNGS enables detection of low fungal loads, making the distinction between colonization and infection a routine clinical challenge.

### Case report

**Figure 1:**
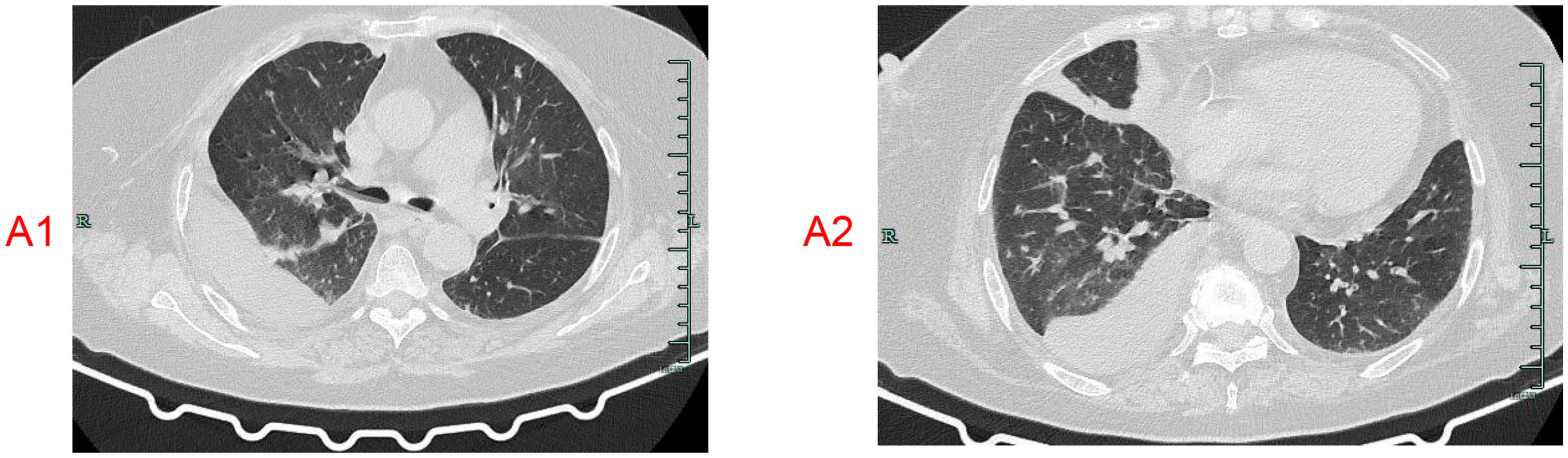
**(A1–E2)** Changes in chest CT during the entire treatment process.

**Figure 2:**
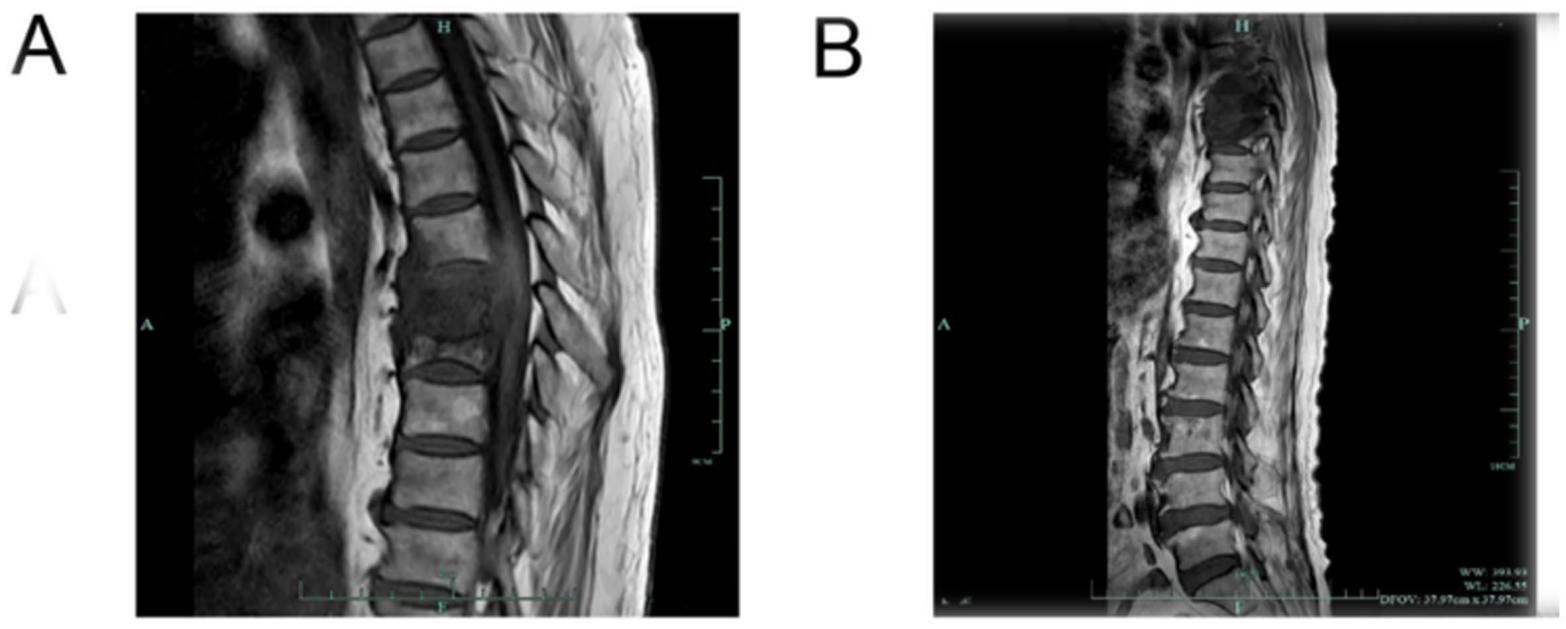
**(A,B)** Results of thoracic MRI before treatment.

A 73-year-old female patient was admitted on January 22, 2024, with a 2-year history of recurrent thoracolumbar pain and fever. Two years prior, she was diagnosed with a thoracic vertebral infection at a local hospital and achieved symptom resolution after treatment with ceftoperazone/sulbactam. Six months before admission (August 2023), symptoms recurred. Chest CT revealed bilateral pulmonary inflammatory lesions, right pleural effusion, mediastinal lymphadenopathy, vascular calcifications (aorta/coronary arteries), and minor pericardial effusion (Figures 1A1,A2). Thoracic MRI demonstrated pathological fractures at T7-T8 vertebrae, bone marrow edema at T6, and degenerative thoracolumbar changes (Figure 2A). Symptoms improved following vancomycin therapy.

Three months prior (October 2023), the patient experienced post-COVID-19 exacerbation with dyspnea. Despite a 15-day course of intermittent steroids (dexamethasone 0.325 mg, oral, twice a day or methylprednisolone 4 mg, oral, twice a day) and antibiotics, symptoms persisted, culminating in acute renal failure requiring hemodialysis.

Five days before admission (January 18, 2024), bronchoalveolar lavage fluid (BALF) mNGS by Hangzhou Genetron Medical Laboratory detected: Pneumocystis jirovecii: 29,036 sequences (21.95% abundance), *Mycobacterium tuberculosis* complex: 2,544 sequences (1.92% abundance).

The patient was initiated on trimethoprim-sulfamethoxazole (SMZ-Co 0.48 g, oral, twice a day) and transferred to a tuberculosis specialty hospital for suspected coinfection management.

**Figure 3:**
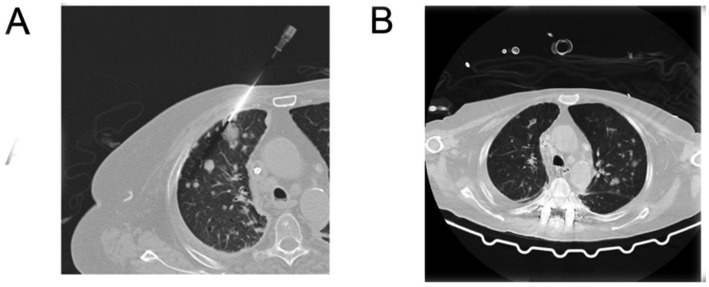
**(A)** Puncture biopsy, lesion area in upper right lung (January 30, 2024), **(B)** right upper lung puncture area after treatment (April 16, 2024).

**Figure 4:**
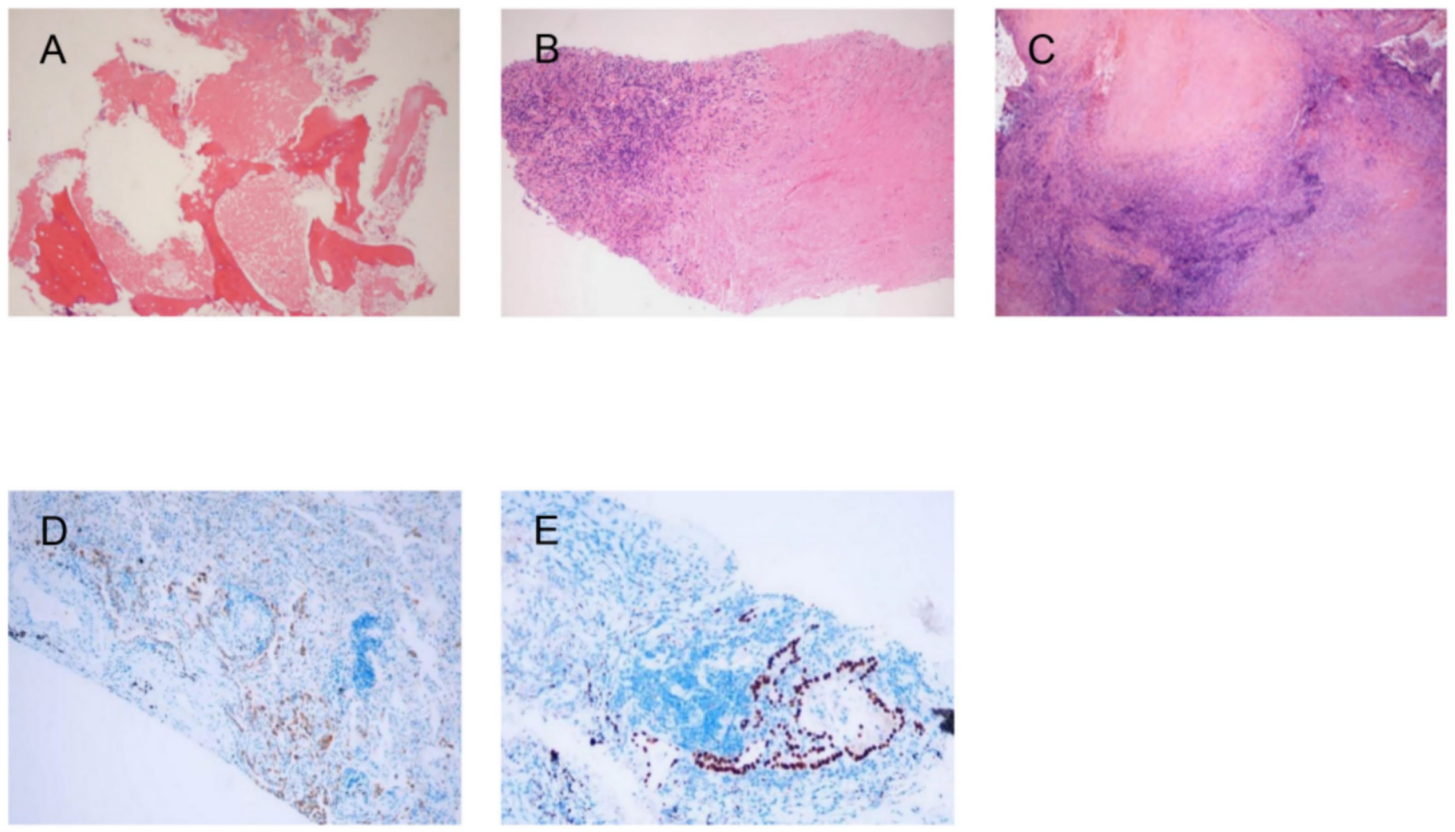
Pathological results **(A)**: T7 vertebral lesion demonstrating osteolytic bone destruction with degenerative necrosis and scattered infiltration of acute and chronic inflammatory cells; Special stains: Congo red (−), acid-fast (−), PAS (−), PAM (−), AB (−). **(B)** Chronic inflammation of pulmonary tissue with extensive coagulative necrosis, surrounded by scant atypical granuloma formation; Immunohistochemistry: TTF-1 (+), CK7 (+), P40 (−), Ki-67 (+ rare); Special stains: Acid-fast (−), PAS (−), PAM (−), AB (−). **(C)** Chronic granulomatous inflammation with necrosis; Special stains: Acid-fast (−), PAS (−), PAM (−), AB (−). Immunohistochemical results **(D)**: TTF-1: Positive (brown chromogen); **(E)** CK7: Positive (brown chromogen).

The patient has a history of hypertension treated with bisoprolol and amlodipine (5 mg, oral, once a day) and diabetes mellitus managed with insulin aspart and glargine. Pre-admission evaluations (December 30, 2023) showed: negative serum HIV antibody, serum (1, 3)-*β*-D-glucan <37.5 pg./mL (negative: <70 pg./mL), Aspergillus galactomannan antigen 0.11 (negative: <0.5), and lymphocytopenia (0.38 × 10^9^/L, January 13, 2024). A thoracic MRI (December 28, 2023, Hangzhou Hospital of TCM) revealed T7-T8 vertebral destruction with abscess (Figure 2B). On January 24, 2024, a C-arm-guided biopsy of the vertebral lesion (Figure 3A) yielded pus and hemorrhagic fluid; subsequent testing confirmed *Mycobacterium tuberculosis* complex (MTBC)-positive, rifampicin-sensitive, and positive MTB DNA/RNA. A lung lesion biopsy (January 30, 2024; Figures 3A,B) and tissue NGS (Hangzhou ADICON Lab) detected overwhelming Mycobacterium dominance (73,211 sequences, 87.07% abundance; MTBC: 72,040, 85.68%). During hospitalization, bilateral lower limb weakness developed (right: Grade 1; left: Grade 3). On February 15, 2024, under general anesthesia, T6-T8 interbody fusion, lesion resection, and pedicle screw fixation were performed, revealing severe osteolysis, necrotic bone, and extensive granulomatous tissue in spinal canals. Necrotic debris and granulomas were excised for histopathology (Figure 4).

**Figure 5:**
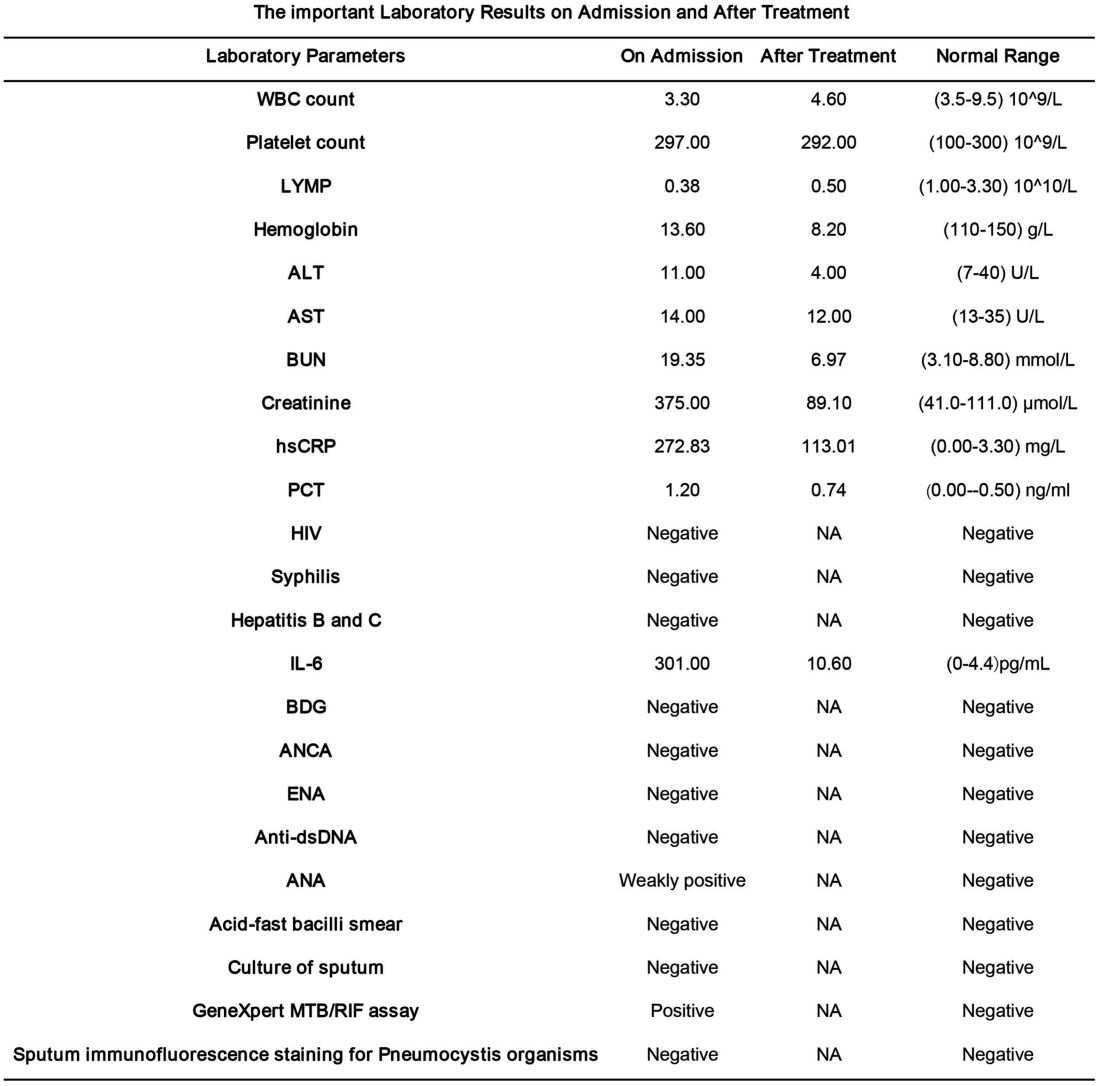
The important laboratory results on admission and after treatment.

**Figure 6:**
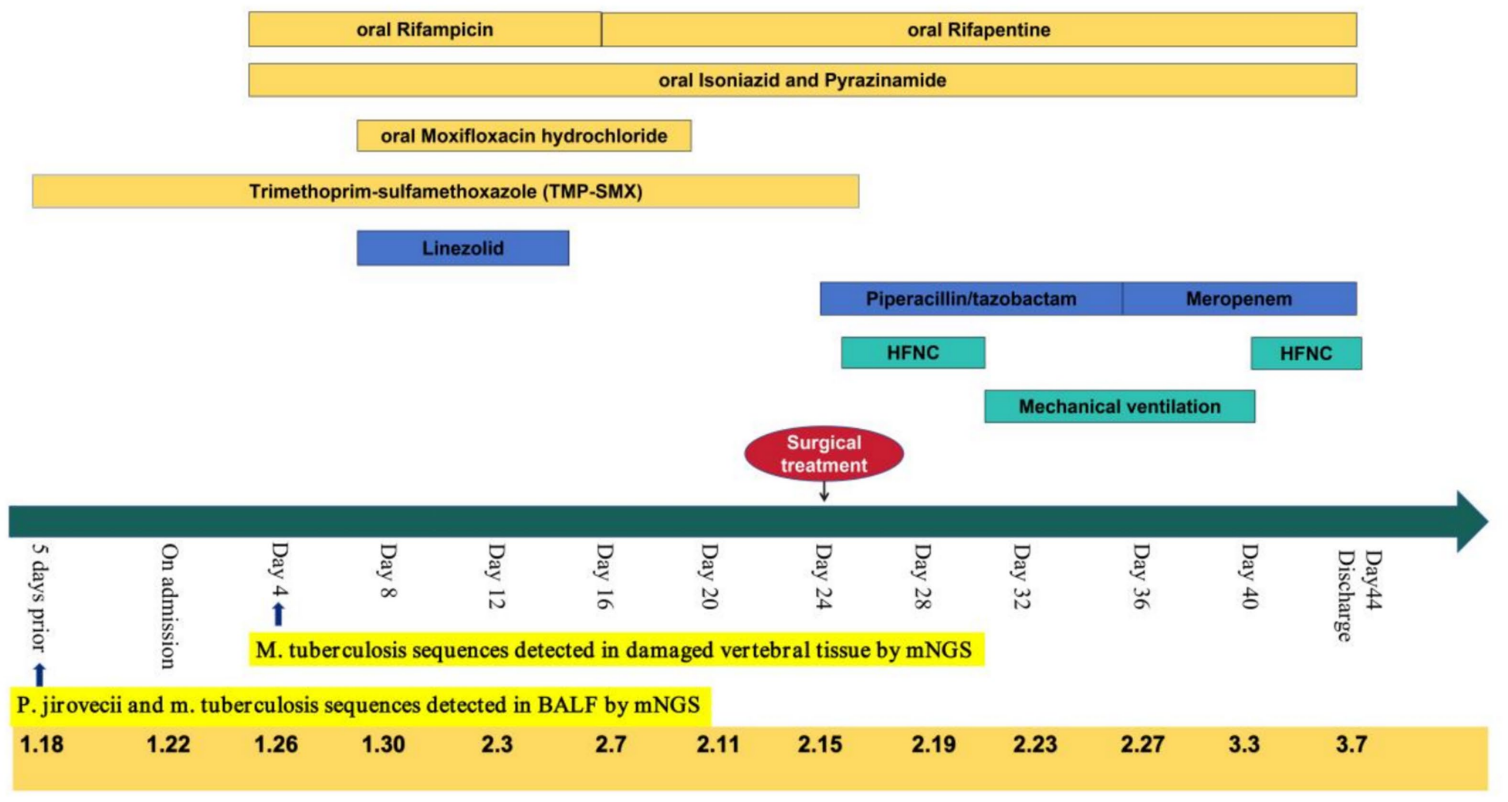
The details of antibiotic and main process of disease.

Following surgery, the patient was transferred to the ICU for monitoring. Postoperatively, she exhibited Grade 1 bilateral lower limb muscle strength, clear consciousness, and impaired cough reflex. Treatment included intensified anti-infective therapy, nutritional support, and physical rehabilitation. On March 3, 2024, the endotracheal tube was removed, and high-flow nasal oxygen with humidification was initiated, though expectoration remained poor. Upon family request, the patient was transferred to another facility on March 7, 2024, where a regimen of rifampicin (0.45 g/day) orally, isoniazid 0.3 g/day, linezolid, oral, twice a day, and post-dialysis amikacin (standard dosing) was continued. Subsequent pulmonary improvement was observed (Figure 3). During hospitalization, anti infection and anti tuberculosis measures were administered (Figure 5), as well as changes in relevant indicators before and after treatment (Figure 6).

BALF Metagenomic NGS Results (April 2, 2024 | Hangzhou Jieyi Medical Laboratory).

Dominant Pathogens:

Klebsiella pneumoniae: 64,274 sequences (31.40% abundance),

Klebsiella spp.: 85,291 sequences (41.65% abundance),

Elizabethkingia anophelis: 13,244 sequences (6.47% abundance).

Additional Findings:

*Mycobacterium tuberculosis* complex: 279 sequences (0.15% abundance),

Staphylococcus aureus: 487 sequences (0.24% abundance),

Pseudomonas aeruginosa: 49 sequences (0.03% abundance),

Human herpesvirus 5 (CMV): 2,799 sequences (1.38% abundance).

## Discussion and conclusion

Pneumocystis jirovecii and *Mycobacterium tuberculosis* frequently manifest as opportunistic pulmonary pathogens in patients afflicted with (HIV). However, their concurrent infection remains an exceptionally rare occurrence in HIV-seronegative individuals. A meticulous comparative analysis of the lung disease spectrum, conducted through the examination of BALFfrom HIV-infected (*n* = 1768) and non-HIV-infected (*n* = 443) individuals, unveiled that CMV infection (20.9%) was the most prevalent condition in the HIV-infected cohort. This was followed by infections caused by P. jirovecii (13.0%), fungi (3.5%), and mycobacteria (3.4%), with *Mycobacterium tuberculosis* accounting for 3.1% of the cases. It is noteworthy that instances of double infections (17.0%, 300/1768) and triple infections (0.6%, 10/1768) were exclusively observed in the HIV-infected population (1).

In our case, the patient had a more than 2-year history of thoracic vertebral infection. Five months prior to admission, magnetic resonance imaging revealed thoracic vertebral destruction. The patient experienced shortness of breath due to COVID-19 infection, which did not improve with treatment. CT scans of the lungs showed multiple nodules and bilateral pleural effusion with thickening. Five days before the current transfer, next-generation sequencing of BALFdetected Pneumocystis jirovecii and *Mycobacterium tuberculosis*, with a sequence count of 29,036 for P. jirovecii. The patient’s bone destruction, nodular lung changes, tuberculosis-related tests on thoracic lesion pus after hospitalization, and NGS results from lung nodule tissue all supported the diagnosis of thoracic and pulmonary tuberculosis. The 10-day TMP-SMX treatment administered before the lung biopsy may have suppressed any potential PJP infection, and we consider the probability of this happening to be extremely low. However, the question remains whether the patient also had a concurrent P. jirovecii infection. It is known that P. jirovecii infections commonly occur in immunocompromised individuals, such as those with HIV, leukemia, or undergoing chemotherapy, but are rare in HIV-seronegative patients. Concurrent infections with P. jirovecii and *M. tuberculosis* are even more uncommon, necessitating cautious interpretation of test results by clinicians. After analyzing four articles on concurrent P. jirovecii and *M. tuberculosis* infections in non-HIV patients, we found that three of the patients had a history of long-term corticosteroid use, suggesting that prolonged glucocorticoid therapy is a major risk factor for Pneumocystis pneumonia PCP in HIV-negative patients (2–5).

The risk of developing PCP correlates positively with both the dosage and duration of corticosteroid administration. Guidelines from the National Comprehensive Cancer Network for preventing and treating cancer-related infections recommend considering PCP prophylaxis for individuals receiving at least 20 mg of prednisone equivalent daily for 4 weeks or longer. Typically, patients are diagnosed with PCP while undergoing steroid tapering, with a median maximum daily dose of 80 mg of prednisone over a median duration of 3 months (6). A decade-long multicenter retrospective observational study revealed that prolonged corticosteroid use prior to a PCP diagnosis is independently associated with an increased 90-day mortality rate among HIV-negative patients, especially those with immune-mediated inflammatory diseases (7). In this particular case, the patient had only taken low-dose dexamethasone or methylprednisolone tablets orally for a mere 2 weeks, a regimen unlikely to significantly impair their immunity. Nonetheless, following a COVID-19 infection, the patient’s lymphocyte count remained persistently low. Based on initial mNGS results of lung lavage fluid, the doctor administered cotrimoxazole tablets for 10 days, yet there was no notable improvement in clinical symptoms or CT imaging. Consequently, treatment was discontinued after examining the pathology of lung nodules and mNGS results of local tissue, both of which were negative for Pneumocystis jirovecii.

The characteristic radiological manifestations of Pneumocystis jirovecii pneumonia (PJP) encompass bilateral ground-glass opacities, which may or may not be accompanied by cyst formation. In the diagnostic and therapeutic course of this patient, the lung CT scans did not reveal the typical ground-glass infiltrates, but instead demonstrated pleural effusion and thoracic vertebral bone destruction (8, 9). Thus, the existence of P. jirovecii infection within the lungs remains debatable. Notably, a subset of atypical P. jirovecii pneumonias can exhibit granulomatous nodules PCP on CT imaging, usually manifesting as enlarged, solitary pulmonary nodules. There are also documented cases of disseminated granulomatousmimicking miliary tuberculosis (10). Consequently, lung biopsy retains its status as the gold standard for the definitive diagnosis of Pneumocystis jirovecii granulomatous pneumonia.

Clinically, many patients do not have the conditions for lung tissue biopsy, necessitating a comprehensive analysis by physicians based on diagnostic techniques. In adult hematological patient populations, current guidelines recommend real-time PCR and immunofluorescence (IF) testing of alveolar lavage fluid for patients with suspected Pneumocystis jirovecii infection. A positive result for both tests indicates the highest probability of PCP, and vice versa. If PCR is positive but IF is negative, clinical and radiological manifestations are required to differentiate between colonization and infection. For low PCR expression, additional (1–3)-*β*-D-glucan (BDG) testing is recommended. A negative PCR with a positive IF is considered technically discordant and raises questions about the quality of either result (11, 12). As Pneumocystis jirovecii is essentially a fungus, serum BDG testing can aid in definitive diagnosis. A meta-analysis including 23 studies reported pooled sensitivity and specificity of 91% (95% CI 87%–94%) and 79% (95% CI 72%–84%), respectively, for PJP serum BDG testing. Sensitivity is higher in HIV-infected patients compared to non-HIV patients, with comparable specificity (13). Low lymphocyte count is also a risk factor for Pneumocystis jirovecii, and PJP typically occurs in T-cell immunosuppressed patients (11). mNGS is highly effective in diagnosing PCP in immunocompromised patients, with higher diagnostic sensitivity than Gomori Methenamine Silver staining (GMS) and serum BDG (100% vs. 15 and 74.5%, *p* < 0.001), but lower diagnostic specificity (91.0%) than GMS (100%), similar to BDG (89.6%) (14).

Differentiating between infection and colonization by Pneumocystis jirovecii is a crucial consideration for clinicians prior to initiating treatment. Pneumocystis jirovecii is ubiquitous in the environment, and infection occurs when the organism’s cysts are inhaled, subsequently transforming into trophozoites that parasitize alveolar epithelial cells and the interalveolar septum. The majority of infections are asymptomatic, and research has demonstrated that colonization by Pneumocystis jirovecii is widespread, affecting children, immunocompetent individuals, immunosuppressed populations, HIV-infected patients, and those with chronic lung diseases. Both smoking and geographical factors can influence the colonization of this species, with the majority of hosts harboring Pneumocystis jirovecii without manifesting symptoms or signs of acute pneumonia (15). In this particular case, despite the patient’s low lymphocyte count, the BDG test was negative. Based on a comprehensive analysis of clinical manifestations, radiological features, and the response to anti-tuberculosis treatment, we postulate that the Pneumocystis jirovecii detected in the initial lung lavage fluid by mNGS was in a colonized state. Nonetheless, we cannot definitively rule out the possibility of sample contamination.

## Conclusion

The diagnosis and treatment of this case highlight that mNGS, as a robust pathogen detection tool, provides a rapid approach for early identification of Pneumocystis jirovecii. However, mNGS testing of specimens alone cannot distinguish between pathogen colonization and active infection. Therefore, clinicians should exercise cautious interpretation of laboratory findings.

## Data Availability

The original contributions presented in the study are included in the article/supplementary material, further inquiries can be directed to the corresponding author.
